# Exosomes from LPS-Stimulated hDPSCs Activated the Angiogenic Potential of HUVECs In Vitro

**DOI:** 10.1155/2021/6685307

**Published:** 2021-04-15

**Authors:** Xiangyu Huang, Wei Qiu, Yuhua Pan, Jianjia Li, Zhao Chen, Kaiying Zhang, Yifei Luo, Buling Wu, Wenan Xu

**Affiliations:** ^1^Department of Stomatology, Nanfang Hospital, Southern Medical University, School of Stomatology, 1838 Guangzhou Avenue North, Guangzhou 510515, China; ^2^Shenzhen Stomatology Hospital (Pingshan), Southern Medical University, 143 Dongzong Road, Pingshan District, Shenzhen 518118, China

## Abstract

**Background:**

Exosomes from human dental pulp stem cells (hDPSCs) were indicated to play a positive role in vascular regeneration processes. But the angiogenic capabilities of exosomes from inflammatory hDPSCs and the underlying mechanism remain unknown. In this study, the inflammatory factor lipopolysaccharide (LPS) was used to stimulate hDPSCs, and exosomes were extracted from these hDPSCs. The proangiogenic potential of exosomes was examined, and the underlying mechanism was studied.

**Method:**

Exosomes were isolated from hDPSCs with or without LPS stimulation (N-EXO and LPS-EXO) and cocultured with human umbilical vein endothelial cells (HUVECs). The proangiogenic potential of exosomes was evaluated by endothelial cell proliferation, migration, and tube formation abilities in vitro. To investigate the proangiogenic mechanism of LPS-EXO, microRNA sequencing was performed to explore the microRNA profile of N-EXO and LPS-EXO. Gene Ontology (GO) analysis was used to study the functions of the predicted target genes. Kyoto Encyclopedia of Genes and Genomes (KEGG) pathway analysis was used to estimate the signaling pathways associated with the inflammation-induced angiogenesis process.

**Result:**

Compared to the uptake of N-EXO, uptake of LPS-EXO activated the angiogenic potential of HUVECs by promoting the proliferation, migration, and tube formation abilities in vitro. The mRNA expression levels of vascular endothelial growth factor (VEGF) and kinase-insert domain-containing receptor (KDR) in the LPS-EXO group were significantly higher than those in the N-EXO group. MicroRNA sequencing showed that 10 microRNAs were significantly changed in LPS-EXO. Pathway analysis showed that the genes targeted by differentially expressed microRNAs were involved in multiple angiogenesis-related pathways.

**Conclusion:**

This study revealed that exosomes derived from inflammatory hDPSCs possessed better proangiogenic potential in vitro. This is the first time to explore the role of exosomal microRNA from hDPSCs in inflammation-induced angiogenesis. This finding sheds new light on the effect of inflammation-stimulated hDPSCs on tissue regeneration.

## 1. Introduction

Stem cell-based dental pulp regeneration has been considered as a novel approach for the treatment of inflammatory pulp tissue. However, revascularization in pulpal tissue remains the greatest challenge in biomimetic pulp regeneration. As the vascular system reconstruction is a prerequisite for nutrient and oxygen transportation, angiogenesis plays a fundamental role in pulp regeneration. Angiogenesis is a complex, dynamic process involving several important steps. These steps include endothelial cell proliferation, migration, tube formation. Subsequently, these tubes maturate into functional blood vessels [1].

hDPSCs are a type of mesenchymal stem cell (MSC) with excellent pluripotency and proliferation potential [2]. hDPSCs can be separated conveniently and noninvasively from extracted teeth. Located in a neurovascular niche, hDPSCs have strong potential for neurogenesis and angiogenesis [3]. Many studies have shown that hDPSCs perform their proangiogenic function by guiding endothelial cells [4]. The conditioned medium, secretome, extracellular vesicles, and cytokines from hDPSCs were proven to promote endothelial cell migration and tubulogenesis, and these findings indicated the importance of the paracrine mechanism in the revascularization process [5, 6]. In response to inflammatory stimulation, immunoregulation and regenerative events could be induced by hDPSCs. In these cases, hDPSCs show great clinical value in dental pulp repair and regeneration.

hDPSCs exhibit strong regeneration potential in controlled inflammatory microenvironments, and this potential includes strong differentiation potency and great cellular proliferation, migration, and homing abilities [7]. A similar phenomenon was observed in terms of angiogenesis. Increased blood vessel density was observed in pulpal tissues from deep caries and pulpitis [8]. In response to stimulation with lipopolysaccharides (LPS), the vascular endothelial growth factor (VEGF) expression could be induced in hDPSCs via mitogen-activated protein kinase (MAPK) signaling [9]. However, the mechanism of the proangiogenic effects of inflammatory hDPSCs remains unclear.

The exosome, a crucial element in paracrine mechanisms, is an important means of intercellular communication [10]. As a type of extracellular vesicle (EVs) with a diameter of 30-200 nm, exosomes display favorable safety and stability. Exosomes can migrate in certain directions. The complex cargo contained in exosomes can reflect the state of the parental cells [11]. All these advantages make exosomes a promising cell-free therapeutic tool for regeneration. MSC-derived exosomes display regulatory functions via mRNA, microRNA, and protein transfer [12]. It has been proven that the angiogenesis of target cells can be regulated by microRNAs from exosomes [13]. Xian et al. showed that exosomes from dental pulp cells could promote the proliferation, cytokine expression, and tube formation of human umbilical vein endothelial cells (HUVECs) via p38 MAPK signaling [6]. Interestingly, EVs secreted by inflammatory hDPSCs showed a superior ability in new vessel formation and cutaneous wound healing compared to EVs secreted by healthy teeth. Taken together, these results raised the question of whether exosomes from inflammatory hDPSCs contribute to improved angiogenic ability [14]. In this study, we hypothesized that exosomes derived from hDPSCs from the inflammatory environment have stronger proangiogenesis effects, and these properties are mediated by specific exosomal microRNAs.

In this study, exosomes derived from LPS-stimulated hDPSCs were isolated and characterized. The proangiogenic potential of the exosomes was studied by evaluating the endothelial cell proliferation, migration, and tube formation abilities in vitro. Besides, microRNA expression profiles of LPS-stimulated hDPSC-derived exosomes were analyzed to elucidate the role of microRNAs and the underlying mechanism.

## 2. Materials and Methods

### 2.1. Histological Study

Third molars from healthy human donors (aged 18-24 years) were extracted and collected at the Department of Oral and Maxillofacial Surgery, Nanfang Hospital, Guangzhou, China. This study was approved by the Ethics Committee of Nanfang Hospital, Southern Medical University. Informed consent was obtained from each patient. Teeth in the control group were healthy teeth without periodontitis, caries, pulpitis, or odontalgia. Teeth in the deep caries group were teeth with caries lesions close to the pulp cavity (≤2 mm) but without spontaneous pain. Dental pulp tissues of healthy and deep caries teeth were collected, dissected, and fixed in 4% paraformaldehyde overnight, dehydrated, and embedded in paraffin. Sections for histological analysis were rehydrated and stained with hematoxylin-eosin. For the immunohistochemical staining, samples were incubated in 4°C overnight with primary antibodies CD31(ZM-0044, ZS Bio, Beijing, China) and CD63 (ZM-0288, ZS Bio, Beijing, China) and subsequently incubated with secondary antibodies (Ab6721 Abcam, UK) in 37°C, followed by color development with 3,3′-diaminobenzidine (DAB) staining.

### 2.2. hDPSC Isolation, Culture, and Identification

Third molars without periodontitis or caries from healthy human donors (aged 18-24 years) were extracted, and the pulp tissues were digested to isolate hDPSCs [15]. Subsequently, the hDPSCs were cultured in Dulbecco's modified Eagle's medium (DMEM; Gibco, Grand Island, NY, USA) supplemented with 10% fetal bovine serum (FBS; Gibco, Grand Island, NY, USA), 100 U/mL penicillin, and 100 mg/mL streptomycin (HyClone, NY, USA), in a 5% CO_2_ atmosphere at 37°C. Flow cytometry (Becton Dickinson, Tokyo, Japan) was conducted to identify stem cell surface markers. Passage 3 hDPSCs were suspended at a final density of 5 × 10^5^ cells/mL and incubated with conjugated human antibodies, including CD29-PE, CD90-APC, CD34-PE, CD45-FITC, and CD44-FITC (BD Pharmingen, Franklin Lakes, NJ) in the dark for 1 hour at 4°C. After washing with phosphate-buffered saline (PBS; Corning, NY, USA), the cells were subjected to flow cytometric analysis.

### 2.3. Multilineage Differentiation Assay

Osteogenic and adipogenic inductions were performed to determine the multilineage differentiation potential of the hDPSCs. Passage 3 hDPSCs were cultured in 6-well plates for 14 days. In the osteogenic induction group, 100 nM dexamethasone, 10 mmol/L *β*-glycerophosphate, and 50 mg/mL ascorbic acid (Sigma, St Louis, MO, USA) were added to the culture medium, and the mineralized nodules were stained with 2% Alizarin red S (Alizarin Red S A5533, Sigma-Aldrich). In the adipogenic differentiation group, 1 mmol/L dexamethasone, 0.05 mmol/L methyl isobutyl xanthine, 10 mg/mL insulin, and 200 mmol/L indomethacin (Sigma, St Louis, MO, USA) were added to the culture medium, and the lipid droplets were visualized by oil red O staining following a standard protocol.

### 2.4. Cell Viability Assay

The hDPSCs were seeded in 96-well plates at a density of 2 × 10^3^ cells/well and were stimulated with different concentrations of LPS (Solarbio, Beijing, China; 0, 1, 5, 10, and 50 *μ*g/mL) for 2 days. 10 microliters of cell counting kit-8 reagent (CCK-8; Beyotime Biotechnology, Shanghai, China) was added to each well. After 2 hours of incubation in the dark, the absorbance was measured at a wavelength of 490 nm using a microplate reader (BioTek, Swindon, UK). The proliferation ability of HUVECs was also tested by the CCK-8 assay described above. Triplicate repeats were used in this assay.

### 2.5. Exosome-Free Serum Preparation and Exosome Collection

Fetal bovine serum was diluted in DMEM to 20%. Overnight ultracentrifugation at 100,000 g was performed to eliminate the serum-derived exosomes [16]. After reaching 70% confluence, hDPSCs (passages 3 to 5) were cultured in DMEM containing 10% exosome-free bovine serum and 1% penicillin-streptomycin with or without 5 *μ*g/mL LPS for 2 days. The culture medium was collected for exosome purification by programmed centrifugation. The culture medium was centrifuged at 300 × g for 10 min, and the supernatant was harvested for another centrifugation at 2,000 × g for 10 min and subsequently followed by 10,000 × g for 30 min. To purify the exosomes, the supernatants were ultracentrifuged (Optima XPN-100, Beckman Coulter, USA) at 100,000 × g for 70 min. The sedimentary pellet was resuspended in phosphate-buffered saline (PBS) and then ultracentrifuged at 100,000 × g for another 70 min. The exosome pellet was resuspended in 20 *μ*L PBS and stored at -80°C.

### 2.6. Exosome Identification and BCA Protein Assay

The protein concentration of the exosomes was quantified with a Micro BCA Protein Assay Kit (Thermo Fisher, USA). Transmission electron microscopy (TEM) was used to identify the exosome morphology. The exosomes were pipetted onto formvar/carbon-coated TEM grids at room temperature. After staining with 4% uranyl acetate, images of the exosomes were captured by TEM (JEM-1400 PLUS, Tokyo, JAPAN). The particle diameter was determined by nanoparticle tracking assay (NTA) with a NanoSight NS300 (Malvern, Worcestershire, UK). The exosomal surface markers CD9 and CD63 and heat shock protein 70 (HSP70) (System Biosciences, PA, USA) were examined using automated Western blotting. Triplicate repeats were used in this assay.

### 2.7. Exosome Uptake Assay

PKH-67 (0.4 *μ*L, Sigma-Aldrich, St Louis, MO) was added to 200 *μ*L Diluent C and incubated with 20 *μ*L exosomes for 2 min at room temperature. Then, 200 *μ*L exosome-free FBS was added to terminate the reaction. The exosomes were washed in PBS and ultracentrifuged at 100,000 × g for 70 min. HUVECs were cultured in an endothelial growth medium-2 bullet kit (EGM-2; Lonza CC-3162, MD, USA) at 37°C with 5% CO_2_. PKH-67-labeled exosomes were added and incubated for 4 hours at 37°C. The HUVECs were fixed with 4% paraformaldehyde for 20 min. Antifade Mounting Medium with 4′,6-diamidino-2-phenylindole (DAPI; Beyotime Biotechnology, Shanghai, China) was used for nuclear staining. The images of the exosome uptake by HUVECs were captured with an electric inverted microscope (Olympus, Tokyo, Japan).

### 2.8. Proliferation Assay

To evaluate the effect of exosomes on HUVEC proliferation, HUVECs were seeded in 96-well plates at a density of 4 × 10^3^ cells/well. Exosomes derived from normal hDPSCs (N-EXO) or exosomes derived from LPS-stimulated hDPSCs (LPS-EXO) were added (100 *μ*g/mL). Cells were cultured for 1, 3, 5, and 7 days. Cell Counting Kit-8 (Beyotime Biotechnology, Shanghai, China) was added into each well. After 2 hours of incubation in the dark, the absorbance was measured at a wavelength of 490 nm using a microplate reader (BioTek, Swindon, UK). Triplicate repeats were used in this assay.

### 2.9. Migration Assay

A Transwell assay was used to estimate the migration ability of HUVECs in response to N-EXO or LPS-EXO. HUVECs were resuspended into the serum-free culture medium and were lately seeded at a density of 5 × 10^4^ cells/well in the upper chamber of 24-well Transwell plates (Corning, NY, USA). The fresh culture medium with N-EXO or LPS-EXO (100 *μ*g/mL) was added into the lower chamber. An equal volume of PBS was added to the control group. After 24 hours, the cells on the upper surface of the upper chamber were removed, while the migrated cells on the lower surface of the upper chamber were fixed with 4% paraformaldehyde for 20 min. The fixed cells were stain in the 1% crystal violet for 20 min. Four views were chosen randomly from each well, and images were captured by a microscope. The number of migrated cells was calculated and analyzed by ImageJ software. Triplicate repeats were used in this assay.

### 2.10. Tube Formation Assay for Angiogenesis

Tube formation of HUVECs is the critical step of angiogenesis. The Matrigel tube formation assay was conducted to detect the proangiogenic effect of LPS-EXO on HUVECs. HUVECs were pretreated with N-EXO or LPS-EXO (100 *μ*g/mL) for 24 hours. An equal volume of PBS was added to the control group. HUVECs were resuspended, seeded onto Matrigel-precoated (150 *μ*L) (BD Biosciences, San Jose, CA) 48-well plates at a density of 10^5^ cells/well, and incubated at 37°C for 1 to 9 hours. Exosomes or PBS was added to each well. Images of tube formation were obtained with the microscope. The indexes of tube formation were analyzed by ImageJ software. Triplicate repeats were used in this assay.

### 2.11. MicroRNA Sequencing

A total of 3 *μ*g RNA was extracted from each exosome sample and sent to Novogene Co., Ltd. (Beijing, China) for the construction of a small RNA library. After cluster generation, the libraries were sequenced on an Illumina HiSeq 2500 platform (Illumina, CA, USA), and 50 bp single-end reads were generated. A *P* value of 0.05 was set as the threshold for significant differential expression by default. Differentially expressed microRNAs were analyzed. The microRNA target genes were predicted by two bioinformatics tools (miRanda and RNAhybrid). Gene Ontology (GO; http://geneontology.org/) enrichment analysis was used to define gene attributes in organisms from three fields: biological processes (BP), cellular components (CC), and molecular functions (MF) (*P* < 0.05 was used). KOBAS software was used to test the statistical enrichment of the target gene candidates in the Kyoto Encyclopedia of Genes and Genomes (KEGG) pathway database (KEGG; https://www.genome.jp/kegg/pathway.html).

### 2.12. Quantitative Reverse-Transcription Polymerase Chain Reaction (qRT-PCR)

To define the appropriate LPS concentration, hDPSCs were treated with LPS (0, 1, and 5 *μ*g/mL) for 24 hours. To explore the impact of LPS-EXO on the angiogenesis-related gene, HUVECs were treated with N-EXO or LPS-EXO (100 *μ*g/mL) for 24 hours, and the equal volume of PBS was added as a control. The mRNA was extracted from cells by the RNA Isolator Total RNA Extraction Reagent (Vazyme Biotech Co., Ltd., Nanjing, China). Total RNA was reverse transcribed into cDNA using a HiScript II 1st Strand cDNA Synthesis Kit (Vazyme Biotech Co., Ltd., Nanjing, China). qRT-PCR was performed by the SYBR-Green PCR kit (Vazyme Biotech Co., Ltd., Nanjing, China) according to the manufacturer's instructions on a QuantStudio 5 system (Thermo Fisher Scientific, Waltham, MA, USA). The glyceraldehyde-3-phosphate dehydrogenase (GAPDH) was used as the internal controls. The sequences of the mRNA primers are listed as follows. GAPDH: forward, 5′-GGACACTGAGCAAGAGAGGC-3′, and reverse, 5′-TTATGGGGGTCTGGGATGGA-3′. Interleukin-6 (IL-6): forward, 5′-AGGAGACTTGCCTGGTGAAA-3′, and reverse, 5′-CAGGGGTGGTTATTGCATCT-3′. Tumor necrosis factor-alpha (TNF-*α*): forward, 5′-CTATCTGGGAGGGGTCTTCC-3′, and reverse, 5′-GGTTGAGGGTGTCTGAAGGA-3′. VEGF: forward, 5′-AGGGCAGAATCATCACGAAGT-3′, and reverse, 5′-AGGGTCTCGATTGGATGGC-3′. Kinase-insert domain-containing receptor (KDR): forward, 5′-TACGTTGGAGCAATCCCTGT-3′, and reverse, 5′-TACACTTTCGCGATGCCAAG-3′. Angiopoietin 1 (Ang-1): forward, 5′-ACCGTGAGGATGGAAGCCTAGA-3′, and reverse, 5′-AATGAACTCGTTCCCAAGCCAATA-3′. Thrombospondin 1 (THBS): forward, 5′-AGACTCCGCATCGCAAAGG-3′, and reverse, 5′-TCACCACGTTGTTGTCAAGGG-3′. To verify the microRNA sequencing results, the microRNA from N-EXO or LPS-EXO was extracted and reverse transcribed by using a Midetect A Track miRNA qRT-PCR Starter Kit (RiboBio Ltd., Guangzhou, China). qRT-PCR was performed. U6 was used as the internal control for microRNA. The primers for miRNAs were designed by RiboBio Corporation (Guangzhou, China).

### 2.13. Statistical Analysis

Each experiment was repeated in triplicate. All the values are presented as the mean ± SD and were analyzed in SPSS 19.0 (SPSS Inc., USA). A paired *t*-test was used for two-group comparisons. One-way analysis of variance (ANOVA) followed by Dunnett's post hoc test was used for multiple group comparisons. *P* < 0.05 was regarded as statistically significant.

## 3. Results

### 3.1. The Blood Vessel Density and Exosome Expression Increased in Deep Caries Dental Pulp

**Figure 1:**
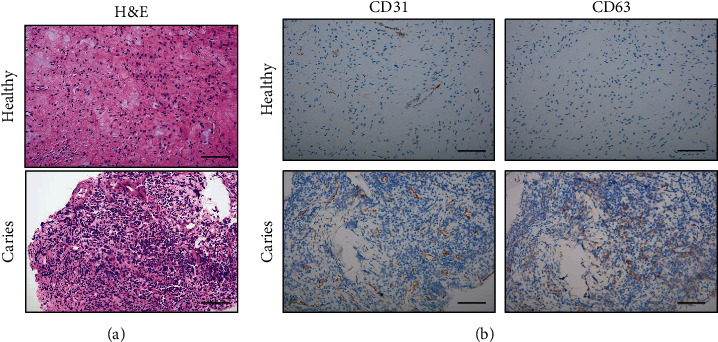
Histological study of the inflammatory dental pulp. (a) H&E stain of dental pulp tissue of healthy and deep caries teeth (*n* = 3) (scale bar, 50 *μ*m). (b) Expression of CD63 (exosome marker) and CD31 (vascular endothelial markers) were detected by IHC assay in dental pulp tissue from healthy and deep caries teeth (*n* = 3) (scale bar, 50 *μ*m). The blood vessel density and exosome expression increased in deep caries dental pulp.

The pulp tissues from healthy and deep caries teeth were collected. The H&E staining was used to detect the pathological changes of the pulp tissue. Compared to the healthy pulp tissue, more plasma cells and lymphocytes and increased blood vessel density were observed in the deep caries pulp tissue, suggesting chronic inflammation condition (Figure 1(a)). To identify the vascular density and exosomes, endothelial marker CD31 and exosome marker CD63 were detected by immunohistochemical staining. As the result showed, the increased levels of CD31 and CD63 were detected in the deep caries dental pulp compared to the healthy dental pulp. Taken together, the result above suggested a possible correlation between increased vascular density and exosome in inflammatory conditions (Figure 1(b)).

### 3.2. Isolation and Characterization of hDPSCs

**Figure 2:**
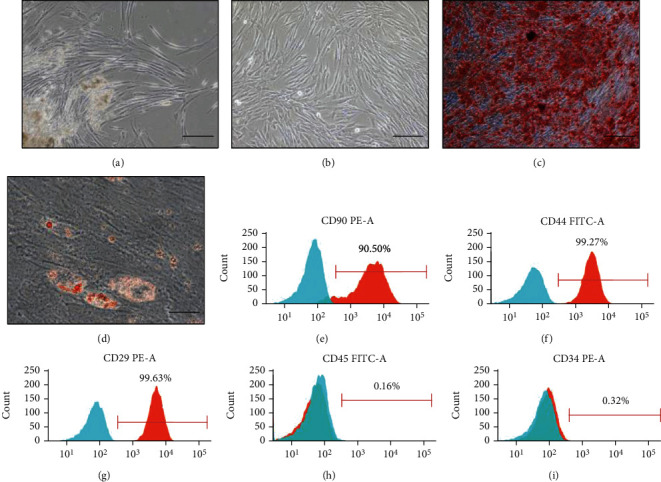
Isolation and identification of hDPSCs. (a) Primary cultured hDPSCs (scale bar, 200 *μ*m). (b) The cell morphology of hDPSCs was observed under an optical microscope (scale bar, 200 *μ*m). (c) Osteogenic differentiation assay of hDPSCs (scale bar, 200 *μ*m). (d) Adipogenic differentiation assay of hDPSCs (scale bar, 50 *μ*m). **(**e–i**)** Flow cytometry assays showed high levels of the mesenchymal stem cell markers CD90 (90.50%), CD44 (99.27%), and CD29 (99.63%) and low levels of the hematopoietic cell markers CD45 (0.16%) and CD34 (0.32%) in hDPSCs.

The hDPSCs were extracted from healthy human third molars. The primary cultured dental pulp stem cells grew around the tissue mass (Figure 2(a)). Morphological observation showed cells with fibroblast-like appearances (Figure 2(b)). To further identify the multilineage-differentiation potential of hDPSCs, the isolated hDPSCs were induced into osteoblasts and adipocytes. The Alizarin red staining showed the formation of mineralized nodules. The lipid droplets were observed in the cytoplasm by oil red O staining (Figures 2(c) and 2(d)). The surface markers of the cells were detected by flow cytometry. The results reflected that the hDPSCs expressed high levels of the mesenchymal stem cell markers CD90 (90.5%), CD44 (99.27%), and CD29 (99.63%) and expressed minimal levels of the hematopoietic cell markers CD45 (0.16%) and CD34 (0.32%), indicating the mesenchymal lineage of hDPSCs (Figures 2(e)–2(i)).

### 3.3. Characterization of Exosomes from LPS-Stimulated hDPSCs

**Figure 3:**
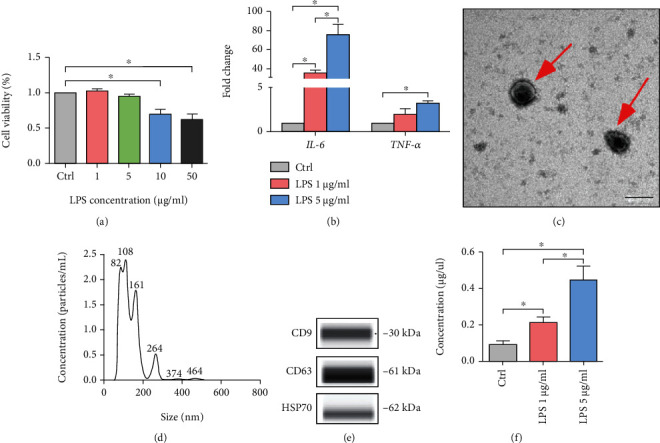
Extraction of exosomes derived from LPS-stimulated hDPSCs. (a) hDPSCs were treated with different concentrations of LPS for 2 days; cell viability was detected by the CCK-8 assay (*n* = 3). (b) The IL-6 and TNF-*α* mRNA expression in LPS-stimulated hDPSCs was detected by qRT-PCR (*n* = 3). (c) TEM showed the classic morphology of exosomes from hDPSCs, which resembled a cup and saucer and had a bilayer membrane (scale bar, 200 nm). (d) The size of the EVs was detected by NTA. (e) The expression of the exosomal surface markers CD9, CD63, and HSP70 was detected by Western blotting. (f) The volume of exosomes secreted from hDPSCs treated with different concentrations of LPS was detected by BCA assay (*n* = 3). Data represent means ± SD. ^∗^*P* < 0.05.

Aiming to establish the inflammatory model in vitro, hDPSCs were stimulated with different concentrations of LPS (0, 1, 5, 10, or 50 *μ*g/mL). The CCK-8 assay showed the effect of LPS on hDPSC viability. No significant difference was observed between the 0, 1, and 5 *μ*g/mL groups. However, compared to that in the control group, the cell viability in the 10 and 50 *μ*g/mL groups decreased to 70% and 62%, respectively (Figure 3(a)). The IL-6 and TNF-*α* expression levels were used as indicators in the in vitro inflammation model. After stimulation with LPS for 24 hours, the IL-6 and TNF-*α* expression levels in the hDPSCs were significantly increased in a dose-dependent manner (*P* < 0.05) (Figure 3(b)). LPS (5 *μ*g/mL) was used as the optimal concentration for stimulation since this concentration could induce an inflammatory microenvironment without reducing cell viability.

Exosomes from hDPSCs were treated with or without LPS stimulation for 2 days and were harvested by programmed ultracentrifugation. TEM was used to detect the shapes of the exosomes. The extracellular vesicles from the hDPSCs presented a typical shape, that is, they were round cup-shaped with a bilayer membrane [17] (Figure 3(c)). To accurately measure the different particle sizes, NTA was used. Most of the exosome diameters ranged from 30 to 200 nm, which was consistent with the standard size of exosomes (Figure 3(d)). Finally, exosome-specific markers (CD9, CD63, and HSP70) were detected by Western blotting (Figure 3(e)). These results indicated that the main content of the purified extracellular vesicles was exosomes.

The BCA assay results showed that the hDPSCs in the LPS-induced inflammatory microenvironment produced more exosomes than those in the normal microenvironment. Furthermore, there was a positive correlation between LPS concentration and exosome volume. The exosome production from 5 *μ*g/mL LPS-treated hDPSCs was higher than that from 1 *μ*g/mL LPS-treated hDPSCs (Figure 3(f)).

### 3.4. Exosomes Derived from LPS-Stimulated hDPSCs Promoted the Proliferation and Migration of HUVECs In Vitro

**Figure 4:**
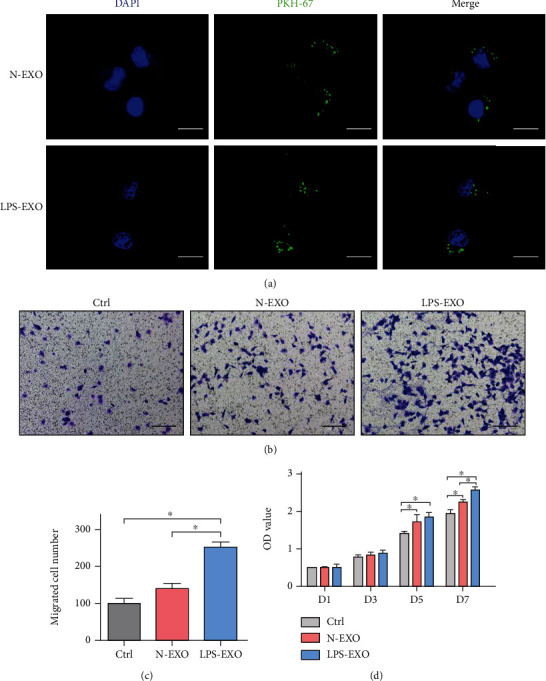
The proliferation and migration of HUVECs were promoted by the uptake of LPS-EXO. (a) Uptake of PKH-67-labeled exosomes by HUVECs (scale bar, 25 *μ*m). (b) Representative images of the Transwell migration assay. LPS-EXO promotes the migration of HUVECs (scale bar, 200 *μ*m). (c) Quantification analysis of the migrated HUVEC number in 24 hours (*n* = 3). (d) The proliferation of HUVECs. LPS-EXO promotes proliferation of HUVECs in 5 and 7 days (*n* = 3). Data represent means ± SD. ^∗^*P* < 0.05.

The N-EXO and LPS-EXO labeled with PKH-67 were uptaken by HUVECs and were mainly located in the cytoplasm (Figure 4(a)). This result indicated that exosomes could be a vehicle for intercellular communication.

To study the effect of LPS-EXO on HUVEC migration, a Transwell assay was conducted. At 24 hours, the HUVECs were observed to migrate through the Transwell membrane in all groups. Compared with the control and N-EXO group, the number of migrated cells in LPS-EXO was significantly increased. The results above revealed that LPS-EXO might have better proangiogenic potential by improving the proliferation and migration of HUVECs (Figures 4(b) and 4(c)).

To investigate the effect of LPS-EXO on HUVEC proliferation, the CCK-8 assay was conducted on 1, 3, 5, and 7 days. The result showed the HUVEC proliferation was not affected by LPS-EXO on 1 and 3 days. But on 5 and 7 days, the proliferation ability of HUVECs was significantly improved by LPS-EXO stimulation when compared to the control and N-EXO group (Figure 4(d)).

### 3.5. Exosomes Derived from LPS-Stimulated hDPSCs Promoted the Tube Formation of HUVECs In Vitro

**Figure 5:**
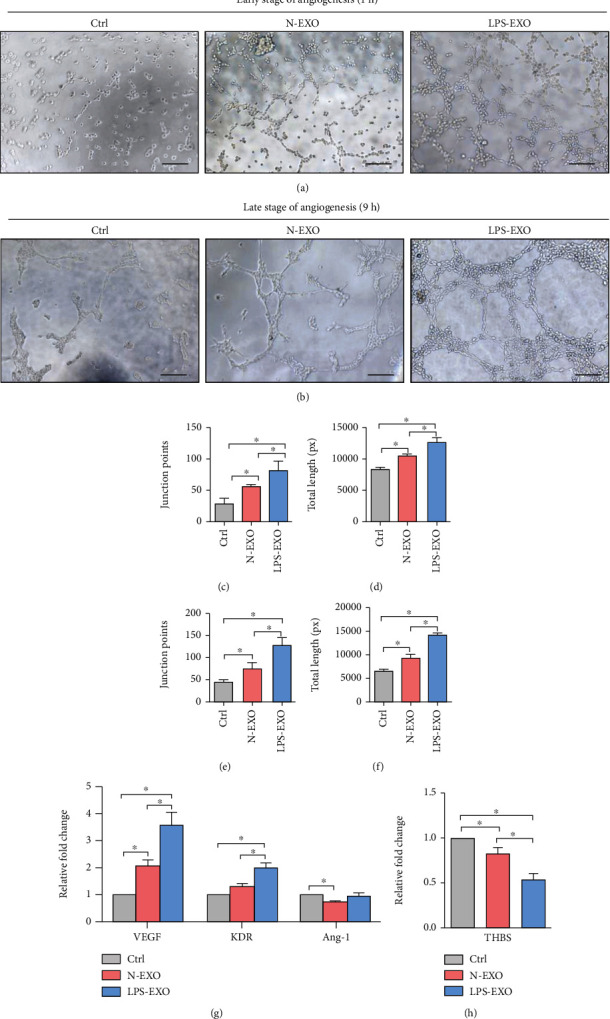
LPS-EXO promotes the angiogenesis of HUVECs. (a) Representative images of the tube formation assay at 1 hour (scale bar, 200 *μ*m). (b) Representative images of the tube formation assay at 9 hours (scale bar, 200 *μ*m). (c, d) Quantification analysis of the number of junction points (c) and total length (d) at 1 hour (*n* = 3). (e, f) Quantification analysis of the number of junction points (e) and total length (f) at 9 hours (*n* = 3). (g) The mRNA expression of proangiogenic genes (VEGF, KDR, and Ang-1) (*n* = 3). (h) The mRNA expression of antiangiogenic genes (THBS). Data represent means ± SD. ^∗^*P* < 0.05.

Tube formation ability of HUVECs was the critical factor for angiogenesis. To investigate the different tube formation effects of N-EXO and LPS-EXO, HUVECs were treated with 100 *μ*g/mL N-EXO or LPS-EXO for 1 hour and 9 hours; the number of junction points and total tube length were analyzed. At the early stage of angiogenesis (1 hour), capillary-like structures begin to form. The chain structures could be observed in all the groups. The total tube length and junction points were higher in the LPS-EXO group than those in the N-EXO group. (*P* < 0.05; Figures 5(a), 5(c), and 5(d)). At 9 hours, at the late stage of angiogenesis, endothelial vessel-like networks had formed in N-EXO and LPS-EXO groups. The LPS-EXO group exhibited the greatest tube structure and had the largest quantity in junction points and total tube length (*P* < 0.05; Figures 5(b), 5(e), and 5(f)).

Besides, the angiogenic process was highly dependent on the balance between proangiogenic and antiangiogenic mediators. In this study, the effect of LPS-EXO on the expression of proangiogenic mRNA and antiangiogenic mRNA was estimated. Compared with those in the N-EXO stimulation group, the VEGF and KDR mRNA levels were upregulated and the THBS mRNA levels were downregulated in the LPS-EXO stimulation group (*P* < 0.05; Figures 5(g) and 5(h)). However, compared to that in the control group, Ang-1 expression was decreased in the N-EXO stimulation group and was not significantly changed in the LPS-EXO stimulation group (*P* > 0.05; Figure 5(g)). The results indicated that LPS-EXO displayed better angiogenesis function than N-EXO.

### 3.6. Differentially Expressed Exosomal MicroRNAs in N-EXO and LPS-EXO

**Figure 6:**
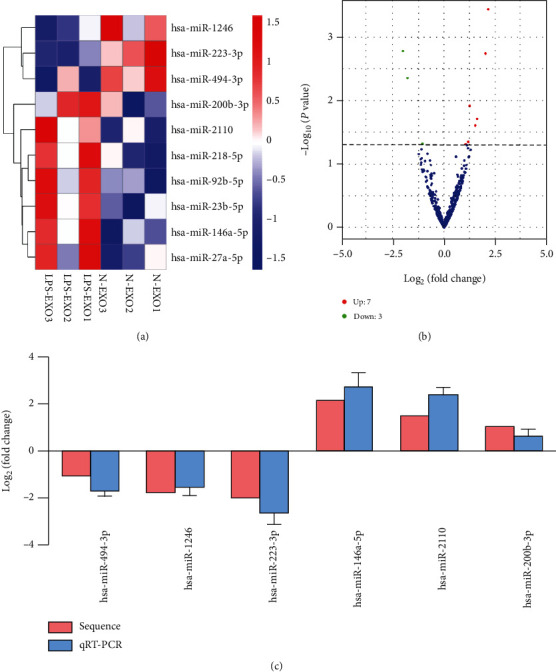
MicroRNA sequencing of N-EXO and LPS-EXO from hDPSCs. (a) Heat map and cluster analysis show that the expression of 10 microRNAs was significantly changed in LPS-EXO, and of these microRNAs, 7 were increased and 3 were decreased. (b) MicroRNA volcano plot. (c) Comparison of microRNA sequencing and qRT-PCR data (*n* = 3). Data represent means ± SD.

To elucidate the different microRNA constituents of N-EXO and LPS-EXO, microRNA sequencing was conducted. The expression of 10 microRNAs was significantly up-/downregulated in the LPS-EXO, and of these microRNAs, 7 microRNAs (miR-146a-5p, miR-92b-5p, miR-218-5p, miR-23b-5p, miR-2110, miR-27a-5p, and miR-200b-3p) were upregulated and 3 microRNAs (miR-223-3p, miR-1246, and miR-494-3p) were downregulated (Figures 6(a) and 6(b)). The qRT-PCR analysis was used to verify the accuracy of the sequencing results. Consistent with the sequencing results, the expression of miR-146a-5p, miR-2110, and miR-200b-3p was upregulated in the LPS-EXO, while the expression of miR-223-3p, miR-1246, and miR-494-3p was downregulated (Figure 6(c)).

### 3.7. Pathway and GO Analysis of Genes Targeted by Differentially Expressed MicroRNAs

The target genes of 10 differentially expressed microRNAs were predicted by 2 bioinformatics tools (miRanda and RNAhybrid). The intersection of the target gene was used for further GO and KEGG analyses.

**Figure 7:**
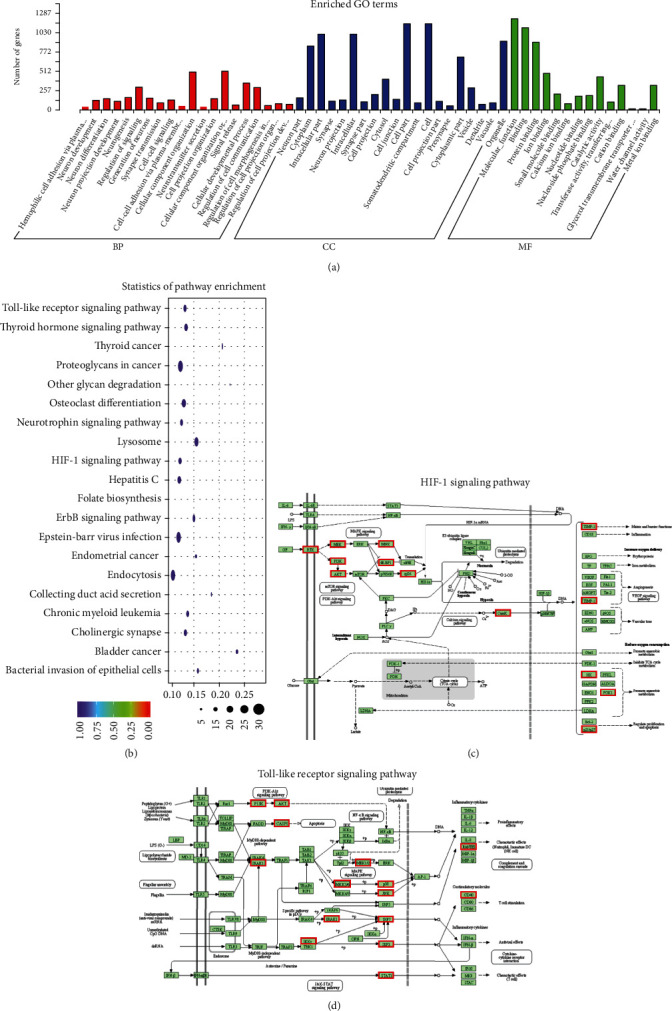
GO annotation and KEGG pathway analysis of differentially expressed microRNAs. (a) GO enrichment analysis of genes targeted by differentially expressed microRNAs. (b) KEGG pathway analysis of differentially expressed microRNAs. (c) HIF-1 signaling pathway map. (d) Toll-like receptor signaling pathway map. Target genes of differentially expressed microRNAs are marked in red.

GO analysis of the target genes showed the most significant biological processes, including cellular component organization, regulation of cellular communication, and cellular development process (Figure 7(a)). KEGG pathway analysis showed that the targeted genes were involved in multiple important signal transductions (Figure 7(b)), including the hypoxia-inducible factor-1 (HIF-1) signaling pathway (Figure 7(c)), thyroid cancer related to angiogenesis, the Toll-like receptor signaling pathway (Figure 7(d)), bacterial invasion of epithelial cells related to inflammation, and endocytosis related to exosome uptake.

**Figure 8:**
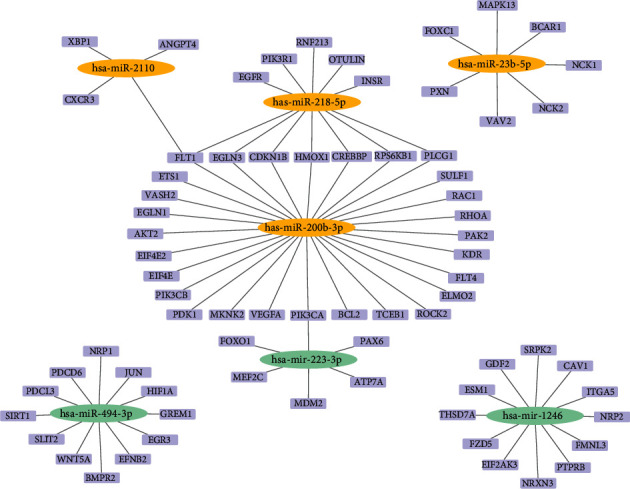
The regulatory networks of microRNAs and angiogenesis-related target genes.

Four different online microRNA databases (TargetScan, miRTarBase, miRDB, and miRWalk) were used to filter the angiogenesis-related genes targeted by the differentially expressed microRNAs. Genes that were indicated as targets by at least 2 of the databases mentioned above were included. Genes were annotated by the DAVID bioinformatics database (https://david.ncifcrf.gov/). According to the GO term analysis, the genes that were related to the biological process of angiogenesis are shown in the mRNA-microRNA network (Figure 8).

## 4. Discussion

Angiogenesis is a prerequisite for and hallmark of dental pulp repair and regeneration [18]. Neovascularization allows regenerative pulp tissue to perform its physiological function by providing oxygen, delivering nutrients, and facilitating immune response. Angiogenesis is a program involving several steps. The ECs are activated by proangiogenic mediators, followed by EC proliferation, migration, and tube formation, and the capillary loops formed [19]. hDPSCs are regarded as reliable candidates for stem cell-based regeneration strategies due to their outstanding proangiogenic ability [20]. The proangiogenic effect of hDPSCs has been proven in vivo and in vitro [21]. However, whether hDPSCs display different proangiogenic abilities in an inflammatory microenvironment remains largely unknown. In a previous study, increased blood vessel density was detected in dental pulp extracted from deep caries [8]. This result indicates that angiogenesis may also take place in response to inflammation [22, 23]. In another study, the vascular network formation of HUVECs was significantly enhanced in a coculture system of hDPSCs and HUVECs with the addition of TNF-*α* [1]. In our research, when stimulated with 5 *μ*g/mL LPS, the hDPSCs displayed stronger angiogenesis-promoting effects on HUVECs than the normal control hDPSCs. Our finding is consistent with the studies mentioned above. We hypothesize that in the early stage of inflammation, hDPSCs may play a protective role in tissue repair by reacting to inflammatory factors and then promote angiogenesis in HUVECs. Further studies are needed to demonstrate how hDPSCs respond to different types of inflammatory factors.

hDPSCs could regulate the function of HUVECs through various kinds of intercellular communication, such as paracrine and juxtacrine communication [24]. As an important component of paracrine, exosomes carry specific biomolecules, including proteins, mRNAs, and microRNAs. It has been widely reported that exosomes play an important role in regulating multiple regeneration processes [25, 26]. In a study by Xian et al., exosomes derived from hDPSCs were shown to promote the angiogenic potential of HUVECs by inhibiting the p38 MAPK signaling pathway [6]. Under inflammatory conditions, exosomes seem to have different capabilities. In another study, when cocultured with exosomes derived from LPS-pretreated hDPSCs, Schwann cells showed better migration and odontoblast differentiation abilities [27]. Furthermore, EVs from periodontitis-hDPSCs exhibited a stronger effect on angiogenesis and wound healing [14]. In our study, we demonstrated that the stronger proangiogenic paracrine activity of inflammation-induced hDPSCs was mediated by exosomes. We also observed that the release of exosomes enhanced with increased LPS concentration. Our study provides strong evidence that exosomes are crucial for stem cell-based regeneration.

The cell signaling pathways by which exosomes from hDPSCs regulate angiogenesis in an inflammatory environment remain unclear. Exosomal microRNAs negatively regulate the expression of their target genes by binding to the 3′UTRs of the target genes, causing translational repression [28, 29]. By conducting microRNA sequencing, we found that the expression of certain microRNAs was downregulated/upregulated in the LPS-EXO. In total, the expression of 10 microRNAs was significantly altered in response to LPS stimulation in our study, and of these microRNAs, 7 microRNAs were increased (miR-146a-5p, miR-92b-5p, miR-218-5p, miR-23b-5p, miR-2110, miR-27a-5p, and miR-200b-3p) and 3 microRNAs were decreased (miR-223-3p, miR-1246, and miR-494-3p). Among microRNAs, 5 microRNAs have been proven to play important roles in inflammation and HUVEC function and angiogenesis. We assume that the differentially expressed exosomal microRNAs might be the reason why inflammation-stimulated hDPSCs display a stronger revascularization role.

miR-223-3p has been confirmed to regulate the function of various systems, including the cardiovascular system and immune system [30]. In head and neck squamous cell carcinoma (HNSCC) tissues, the miR-223-3p expression was negatively correlated with the CD31 expression, indicating its antiangiogenic properties [31]. The mRNA and protein expression of VEGF was significantly increased in breast cancer cells in which miR-223-3p was inhibited in vitro [32]. Furthermore, miR-223 was deregulated in several types of inflammatory diseases, such as sepsis, type 2 diabetes, and rheumatoid arthritis [33]. Taken together, these findings suggest that miR-223-3p is a strong candidate for the mechanism by which the LPS-EXO-derived microRNA promotes the angiogenesis of HUVECs. We also found some other interesting observations by reviewing articles. In the study by Li et al., the expression of miR-146a was induced by LPS treatment. Angiogenesis was inhibited by miR-146a knockdown via TGF-*β*1 signaling pathway activation [34]. In another study, the expression level of miR-218-5p in glomerular mesangial cells (GMCs) was upregulated by LPS stimulation [35]. miR-218-5p knockdown promoted the apoptosis of HUVECs by activating HMGB1 [36]. miR-200b-3p was reported to affect HUVEC functions by directly regulating a variety of proangiogenic genes (e.g., VEGFA) and antiangiogenic target genes (e.g., KLF2) [37]. A previous study revealed that miR-1246 inhibited angiogenesis by repressing NF-*κ*B signaling [38]. Further studies are required to demonstrate how certain LPS-EXO microRNAs promote the angiogenesis of HUVECs.

## 5. Conclusion

In the current study, we found that exosomes from LPS-stimulated hDPSCs displayed a stronger effect on promoting the angiogenesis of HUVECs than exosomes from normal hDPSCs. Our study also showed that the altered expression of certain exosomal microRNAs might be the reason for the enhanced proangiogenic ability of LPS-stimulated hDPSCs. To the best of our knowledge, this is the first study to demonstrate the role of exosomal microRNAs from hDPSCs in inflammation-induced angiogenesis. The current study may shed light on the effect of inflammation-stimulated hDPSCs on tissue regeneration.

## Data Availability

The high-throughput microRNA sequencing data used to support the findings of this study are available from the corresponding author upon request.

## Abbreviations

hPDSCs: Human dental pulp stem cells
HUVECs: Human umbilical vein endothelial cells
MSC: Mesenchymal stem cell
LPS: Lipopolysaccharides
EVs: Extracellular vesicles
N-EXO: Exosomes derived from normal hDPSCs
LPS-EXO: Exosomes derived from LPS-stimulated hDPSCs
FBS: Fetal bovine serum
DMEM: Dulbecco's modified Eagle's medium
PBS: Phosphate-buffered saline
EGM-2: Endothelial growth medium-2
CCK-8: Cell counting kit-8
TEM: Transmission electron microscopy
NTA: Nanoparticle tracking assay
DAPI: 4′,6-Diamidino-2-phenylindole
qRT-PCR: Quantitative reverse-transcription polymerase chain reaction
HSP70: Heat shock protein 70
VEGF: Vascular endothelial growth factor
KDR: Kinase-insert domain-containing receptor
Ang-1: Angiopoietin 1
THBS: Thrombospondin 1
IL-6: Interleukin-6
TNF-*α*: Tumor necrosis factor-alpha
GAPDH: Glyceraldehyde-3-phosphate dehydrogenase
GO: Gene Ontology
KEGG: Kyoto Encyclopedia of Genes and Genomes
BP: Biological processes
CC: Cellular components
MF: Molecular functions
MAPK: Mitogen-activated protein kinase
HIF-1: Hypoxia-inducible factor-1
HNSCC: Head and neck squamous cell carcinoma
GMCs: Glomerular mesangial cells.
